# COVID-19 vaccine confidence project for perinatal women (CCPP)—Development of a stepped-care model to address COVID-19 vaccine hesitancy in low and middle-income countries

**DOI:** 10.3389/fpubh.2022.1100046

**Published:** 2023-01-13

**Authors:** Ramdas Ransing, Pracheth Raghuveer, Aman Mhamunkar, Prerna Kukreti, Manju Puri, Suvarna Patil, Hegde Pavithra, Kumari Padma, Praveen Kumar, Kavya Ananthathirtha, Manish Kumar Goel, Smita N. Deshpande

**Affiliations:** ^1^Department of Psychiatry, BKL Walawalkar Rural Medical College, Ratnagiri, Maharashtra, India; ^2^Department of Epidemiology, National Institute of Mental Health and Neurosciences, Bangalore, India; ^3^Department of Psychiatry, Lady Hardinge Medical College, New Delhi, India; ^4^Department of Obstetrics and Gynecology, Lady Hardinge Medical College, New Delhi, India; ^5^Department of Medicine, BKL Walawalkar Rural Medical College, Ratnagiri, Maharashtra, India; ^6^Department of Community Medicine, Yenepoya Medical College, Yenepoya (Deemed to be University), Mangalore, Karnataka, India; ^7^Department of Paediatrics, Lady Hardinge Medical College, New Delhi, India; ^8^Department of Obstetrics and Gynecology, Kasturba Medical College, Mangalore, Manipal Academy of Higher Education, Manipal, India; ^9^Department of Community Medicine, Lady Hardinge Medical College, New Delhi, India; ^10^Department of Psychiatry, St John's Medical College Hospital, St John's National Academy of Health Sciences, Bangalore, Karnataka, India

**Keywords:** pregnancy, COVID-19, children, vaccine, barriers

## Abstract

**Background:**

COVID-19 vaccine hesitancy (CVH) is common among perinatal women in low and middle-income countries (LMICs), but it is often unaddressed. This could be due to a lack of feasible, scalable, and acceptable interventions and models for CVH in LMICs. Our study aimed to develop a CVH intervention model that can be implemented in LMICs using existing human healthcare resources.

**Methods:**

A literature review was conducted on aspects of vaccine hesitancy, pre-existing interventions, and models for addressing vaccine hesitancy (COVID-19 and non-COVID-19). The lead authors (RR and PKuk) formed a team consisting of vaccinators, experts, and stakeholders. Members shared their perspectives and proposals for various models and interventions that could be implemented in LMICs. A CVH intervention model was developed using a logic model, a WHO implementation toolkit, experts' feedback, and consensus.

**Results:**

A consensus was reached to develop a COVID-19 Vaccine Confidence Project for Perinatal Women (CCPP), which is a primary health care worker (HCWs)-based stepped-care model. The CCPP model includes HCW training, integration into ongoing COVID-19 vaccination programs, CVH screening, CVH intervention, and referral services suitable for implementation in LMICs.

**Conclusion:**

The CCPP project/model provides a practical approach that can help in the early detection and management of CVH. The model can be tailored to different healthcare settings to improve COVID-19 vaccine uptake among perinatal women in LMICs.

## 1. Introduction

The Government of India (GOI) and the World Health Organization (WHO) have recommended several Coronavirus Disease 2019 (COVID-19) vaccines for perinatal women (1, 2). Unvaccinated perinatal women are at a greater risk of COVID-19-related mortality and morbidity, pre-term labor, and fetal death than vaccinated women (2, 3). Still, a substantial proportion of perinatal women are not vaccinated against COVID-19 in low and middle-income countries (LMICs) including India (4).

Vaccination of perinatal women has been regarded as a major strategic weapon against COVID-19. However, COVID-19 vaccine hesitancy (CVH) poses a significant barrier to successful vaccine uptake (4). Vaccine hesitancy is a delay in accepting or refusing vaccination despite its availability (5). Associated factors include lack of information/ misinformation regarding vaccines (related to efficacy, safety, accessibility), low perception of COVID-19 infection, and affordability of vaccines (6, 7).

Addressing CVH requires multi-level interventions: policy and community-based (e.g., reducing the cost of vaccines), organizational (e.g., home visits, reminders, feedback), inter-personal (e.g., recommendations by clinicians to their patients), and individual (e.g., addressing the personal concerns) (8). Presently, the majority of the interventions are policy and community-based, with a primary focus on raising awareness through media. In a few places, vaccination certificates were made mandatory for any travel or employment, but such measures were perceived as an enforced measure (9). Such methods, however, are unethical and likely to increase fear and misconceptions despite the fact that COVID-19 vaccination is effective and beneficial. One reason for implementing such practices could be a lack of individual-level interventions and models to address CVH determinants. Furthermore, capacity building and public health service strengthening for CVH screening and intervention may be insufficient in LMICs, including India.

In this context, we aimed to develop a model of care for addressing CVH in perinatal women which could be feasible, cost-effective, scalable, replicable, applicable, and acceptable and strengthen ongoing efforts of LMIC governments.

## 2. Material and methods

To develop the CVH intervention model/project, first author (RR) invited psychiatrists [*N* = 6: private 3 and government 3)], public health experts [*N* = 4, private 1 and government 3), psychologists [*N* = 2, one each from private and government facilities), obstetricians (*N* = 2, one each from private and government facilities), pediatricians (*N* = 2, one each from private and government facilities), medical officers (government, *N* = 2)], primary health care workers (HCWs) (*N* = 2, one each from private and government facilities), and stakeholders (*N* = 2, one each from private and government facilities), who were actively involved in COVID-19 vaccination drives in hospital or community settings. Group discussions were held through virtual conferencing platforms as the pandemic was active at that time (mainly email, WhatsApp) for 12 weeks (15th August to 15th November 2021). Thereafter an initial plan for the model/project was developed in the following phases.

### 2.1. Phase 1

This phase was directed toward assessing the current scenario of COVID-19 vaccinations and approaches adopted in authors' respective States (Delhi, Maharashtra, and Karnataka) from 15th August to 15th November 2021. The team members discussed about their state specific COVID-19 vaccinations, preparedness plans, and current or future measures implemented or proposed by the Government. They also discussed about the pre-existing infrastructure, ongoing training, and human resources in perinatal health care and COVID-19 vaccinations in the three study sites.

### 2.2. Phase 2

Simultaneously, a literature review was carried out to identify relevant existing information related to CVH, epidemiology, interventions, and recommendations. We appraised: (a) existing systematic reviews on the clinical and psychosocial aspects of vaccine hesitancy (COVID-19 and non-COVID-19) among the general population and perinatal women globally and in India; (ii) epidemiological research on a CVH; (iii) existing interventions and models in India or other countries for CVH. Then, a distillation and matching model was used to facilitate understanding of similarities and differences among interventions or models, to guide intervention/model development, to address gaps in the literature, and to point to possibilities for new interventions/models for CVH for perinatal women in LMICs (10).

### 2.3. Phase 3

The lead authors (RR and PKuk) then created a preliminary conceptual framework based on the existing literature, responses from team members, and challenges in the implementations of strategies using the WHO implementation toolkit and the logic model for the public health (11, 12).

### 2.4. Phase 4

**Figure 1:**
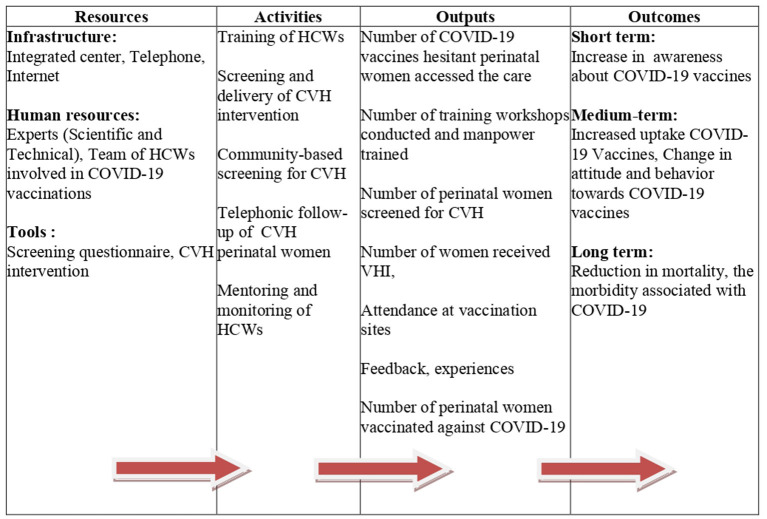
Logic model for the COVID-19 vaccine project confidence project for perinatal women (CCPP model).

**Figure 2:**
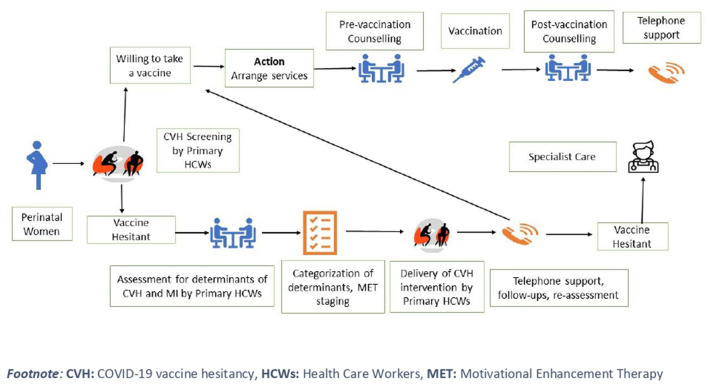
Algorithm for CVH screening and intervention.

A Priori Conceptual Framework was then shared with the team members for feedback, which was then iteratively modified. During the modification phase, team members were asked to provide feedback or comments on the priori conceptual framework and logic model. Using a modified Delphi method (consensus decision making->70 percent representative agreeable), the conceptual frameworks and logic model were revised and approved. The preliminary consensus draft was further discussed with experts (identified by RR and PKuk with predefined criteria) outside the first group (i.e., team members) over 1 month (16th November to 15th December 2021). The Experts (*n* = 4) included a senior professor/consultant with more than 10 years of experience in fields of national immunization program and/or public health/advocacy program for perinatal women, and a background qualification in nursing (*n* = 1), psychiatry (*n* = 2), and public health (*n* = 1). After extensive discussions with experts, a draft of COVID-19 Vaccine Confidence Project for Perinatal Women (CCPP) was prepared and circulated among the team members for final inclusion and approval. The final version of the conceptual framework and logic model (Figures 1, 2) were approved using a modified Delphi method.

### 2.5. Ethics

Approvals from the Institutional Ethics Committees of all the three centers where the model is being implemented and tested: (i) BKL Walawalkar Rural Medical College, Sawarde Maharashtra (BKLWRMC/IEC/589/2021); (ii) Lady Hardinge Medical College, New Delhi (IEC/KMC/MLR 10/2021/309); and (iii) Kasturba Medical College, Mangalore, Karnataka (LHMC/IEC/2021/03/113) were obtained.

## 3. Results

The CCPP model was developed after considering the existing human resources, national priorities for COVID-19 vaccinations, and the possibility of future integration into the national programs. It is a HCWs -based, stepped-care model. It includes screening and delivery of a brief psychosocial intervention for CVH in various health care settings (e.g., community or hospital). The model consists of four components: resources, activities, outputs, and outcomes (Figure 1) which briefly described below.

### 3.1. Resources

#### 3.1.1. Infrastructure

To establish the model in a center, it is important to have attached COVID-19 vaccination services. The infrastructure required includes telephone and internet. This is to ensure that the intervention may be delivered by phone or video calls in rural or remote regions and during public health measures (e.g., lockdowns). Furthermore, phones may be used to send reminders and address queries.

#### 3.1.2. Human resources

The CCPP focuses on developing and implementing the primary HCWs -based care for CVH. Primary HCW-based models have been found to be effective in many LMIC countries for several other health conditions related service delivery, including COVID-19 pandemic times (13–15). Primary HCWs (e.g., Accredited Social Health Activist) are pillars of several national programs, service delivery by them is more acceptable and is better integrated with general healthcare infrastructure. They are more accessible for providing a trusted and reliable source of information to perinatal women and the general public (16). Thus, the CCPP model may be flexible and adaptable across all health care levels.

#### 3.1.3. Tools

Developed CCPP model consist of a screening tool and a CVH intervention. The screening tool consists of two questions: (1) whether you have received any dose of COVID-19 vaccine i.e., current vaccination status and (2) whether you are willing to take COVID-19 vaccine in the next 2 months, if available i.e., willingness to take a second dose of vaccine. Perinatal women who answer “no” to both questions, or “yes” to the first question and “no” to the second, are assessed further for determinants of CVH using standardized self-reported scales (Oxford COVID-19 Vaccine Hesitancy Scale, Oxford COVID-19 Vaccine Confidence & Complacency Scale) in local languages (Marathi, Hindi, and Kannada) (4).

#### 3.1.4. CVH intervention

A brief, individual-level psycho-social intervention was developed using principles of MET (motivational enhancement therapy). A brief outline of our intervention has been published elsewhere (4). Based on stages pf motivation, every perinatal women is classified for CVH into four categories (i.e., pre-contemplation, contemplation, decision, and preparation). The specific determinants of CVH (e.g., confidence, complacency, constraints, calculation, and collective responsibility) are explored using motivational interviewing skills (e.g., asking open-ended questions, using reflective listening, and affirming and reiterating statements) (17) (Figure 2). At present, the CVH intervention is being delivered in different study sites and its outcomes will be reviewed by experts from diverse backgrounds.

### 3.2. Activities

Various activities (Figure 1) that need to be performed for effective implementation are listed.

#### 3.2.1. CCPP training and mentoring

The model was also prepared to train HCWs working in COVID-19 vaccination services for screening and delivery of CVH intervention. This involves didactic lectures, video presentations, self–assessment, group work, and suggested readings, conducted for 4 to 6 hours with fortnightly follow-ups. Also, clinical psychologists and medical officers will provide support as and when required and once weekly for around 15–20 minutes. The training manual is structured and includes details of each session, illustrative case histories, questions and assessment, active listening, and communication skills.

#### 3.2.2. Screening and assessment for CVH

The trained primary HCWs will ask two questions (as mentioned above) to perinatal women at their first contact with maternal and child services [e.g., antenatal (ANC), postnatal (PNC), immunization clinic] or during home visits (Figure 2). Based on results of screening tools and self-reported scales, determinants of CVH will be divided into five categories: confidence, complacency, constraints, calculation, and collective responsibility for delivery of a CVH intervention.

#### 3.2.3. Delivery of CVH intervention

Primary HCWs will deliver the intervention over 25 to 30 minutes *via* telephone (audio or video calls), or in-person. The session is split into two parts of 10–15 minutes each. In the first part, HCWs introduce themselves, establish rapport, and interpret the result of self-reported scales. The HCWs will then educate the perinatal women about the available COVID-19 vaccines and post-vaccination care. A step-wise approach will be adopted to address the CVH determinants using MET techniques. Some of these determinants may be difficult to address due to rapidly evolving evidence about COVID-19 vaccines. In case of any discrepancy, the primary HCWs can refer the perinatal women to specialists such as Psychologist/ Psychiatric Social Worker (Figure 2).

#### 3.2.4. Reminders

Two follow-ups (telephonic, in-person, home visits**)** are to be carried out at a periodic interval of 3 and 6 weeks. During this, the HCW will assess whether the COVID-19 vaccine-hesitant woman has received the vaccine or if there are any additional concerns or myths, they can be addressed. This can serve as a reminder and will help in answering any additional query of perinatal women.

#### 3.2.5. Monitoring

Certain quantitative (e.g., number of perinatal women screened and receiving intervention, mode of delivery of intervention, duration of intervention) or qualitative (e.g., experiences or feedback from both perinatal women and HCWs) performance indicators will be used to monitor intervention delivery.

### 3.3. Outputs and outcomes

Figure 1 depicts the broad categories of outputs and outcomes that would be expected following the implementation of the CCPP model. The expected direct outcomes are (i) increased awareness about COVID-19 vaccination (ii) referrals for CVH intervention with (iii) increase in COVID-19 vaccination coverage for perinatal women. The expected indirect outcomes are (i) adoption of preventive measures, and (ii) reduction of COVID-19 mortality and morbidity.

## 4. Discussion

The CCPP has been developed as a stepped-care model based on primary HCWs and it has five core components: theoretical background, CVH intervention content, intervention delivery, monitoring, as well as training and mentoring model. Published literature suggests that stepped-care model have been proven effective, feasible, acceptable, scalable, and replicable in many LMICs for several medical conditions, including during the COVID-19 pandemic (14, 15, 18).

### 4.1. Integrated center for COVID-19 vaccination and CVH intervention services

Our experts noticed that none of the COVID-19 vaccination centers offer individual-level CVH intervention. Ideally, CVH intervention services should be available at these centers and we intend to provide these services through our model. At these centers, primary HCWs or trained volunteers can screen the perinatal women for CVH. This strategy may result in tangible results and strengthen community participation for COVID-19 vaccination. A recent survey of 44,260 participants in LMICs found that HCWs are the most trusted by service users for COVID-19 vaccine-related information (19). India has a strong workforce of primary HCWs like Junior Health Assistants (JHAs) and Accredited Social Health Activists (ASHAs) who can be utilized effectively in improving the CVH screening and intervention.

At the moment, there is no effective CVH intervention in any of the LMICs. Rather, most perinatal women neither screened for CVH nor do they receive any intervention. In such scenarios, the use of two screening questions in conjunction with CVH scales (e.g., Oxford Covid-19 Vaccine Hesitancy Scale, Oxford COVID-19 Vaccine Confidence & Complacency Scale) has a higher scale-up potential to strengthen the ongoing COVID-19 vaccination drives (4, 20).

The Indian Government started a mass vaccination drive to increase access to COVID-19 vaccines (21, 22). However, due to non-availability of an individual-level interventions for CVH, COVID-19 vaccine-related myths and misconceptions are not adequately addressed. Therefore, a brief psycho-social intervention (e.g., CVH intervention) may help to align the ongoing efforts to improve access to COVID-19 vaccinations. The CVH intervention in our model is based on MET techniques (4). Evidence suggests that MET techniques are useful to reduce vaccine hesitancy (8). This intervention is flexible enough to allow for additional techniques to be added in the future making it more comprehensive and transdiagnostic. Due to ongoing public health measures (e.g., lockdown, isolation), just single face-to-face sessions has been kept.

In India, research on CVH in perinatal women is limited. COVID-19 vaccination experiences, beliefs, and circumstances differ from those of the general population (e.g., apprehension regarding vaccine safety in fetuses, infants). CVH among perinatal women is likely to result in non-vaccination of their children with COVID-19 as well as non-COVID-19 vaccines (23). Moreover, the interventions focused on generating awareness or based on social media are ineffective in addressing individual level determinants of CVH. Feasible and effective individual-level interventions may help in addressing CVH among perinatal women and motivate them to develop positive attitudes toward vaccinations.

Some studies reported that text-based reminders were ineffective to improve the COVID-19 vaccination rates (24). Therefore, we coupled our intervention with telephonic reminders. Experts noticed that HCWs are not advocating the COVID-19 vaccine to perinatal women actively, primarily because the government had not recommended the COVID-19 vaccine to pregnant women during the first phase of COVID-19 vaccinations (25). Moreover, HCWs are not trained to provide any individual-level CVH intervention.

The skills needed to screen for and detect CVH can be learned using our proposed model. This may help primary HCWs to understand the importance of screening for CVH and referring to the nearest COVID-19 vaccination center. Integrated center should maintain a list of HCWs in the region who provide CVH detection and intervention services. This will ensure that the services are provided to the perinatal women in the community. As the COVID-19 situation is dynamic, it is necessary to update the knowledge of HCWs through training. CVH intervention is a living document that can be edited and updated to address the emerging determinants of CVH. Furthermore, trained HCWs can screen and deliver the intervention to other populations (e.g., children and adolescents) and contribute to lowering the overall burden of CVH in the community.

### 4.2. Challenges in the implementation of the CCPP model

Certain challenges expected during the implementation of intervention can be a lack of readiness of the system, limited resources, lower relative priority, lack of incentives, and poor motivation of HCWs. Though providing incentives to HCWs has the potential to increase vaccination coverage, such approaches are unlikely to be sustainable in LMICs. Considering these factors, we developed a brief psycho-social intervention that can be delivered in 25–30 minutes *via* phone or in-person.

### 4.3. Potential benefits of CCPP model

Our model was developed specifically to address CVH through individual-level interventions. During the pandemic, among the various preventive models like public health measures and contact tracing, primary HCWs-based models were successful. Once HCWs with “preventive” focus align with the CCPP concept, this can lead to the launch of new services and improve access to CVH intervention.

Training would strengthen the skills and knowledge of HCWs. Being grass root workers, delivering the intervention would further enhance their presence in the community. It would also increase their professional satisfaction. Further, this model may raise public awareness about COVID-19 vaccinations and, as a result, help to reduce CVH. Moreover, suggested approach is simpler, more cost-effective, sustainable, and easier to integrate into existing programs.

### 4.4. Adapting and using the CCPP model of care in other countries and settings

We attempted to address several key questions related to the implementation with involving primary HCWs, using video conferencing and phone calls. As a result, this model is likely to be more cost-effective, feasible, accessible, scalable, sustainable, replicable, and acceptable for low-resource settings, to be implemented in national programs, and even in pandemic settings. Also, the model is flexible for implementation in government as well as private settings. However, qualitative and quantitative research is needed before local adaptation. Intervention characteristics (single session, transdiagnostic nature, strength of evidence of the content) and implementation processes (e.g., engaging diverse stakeholders) may influence the implementation positively. Thus, the CCPP model may be adapted to other Indian states or LMICs.

### 4.5. Current status of CCPP model in India and future directions

The CCPP model is currently being implemented in India. We have begun training the HCWs at each center. Trained HCWs have reported a considerable increase in knowledge, skill, and confidence in addressing CVH. In upcoming trials, both the model and CVH intervention will be tested for feasibility and effectiveness across three centers. Once the effectiveness has been demonstrated, it can then be further researched or implemented in different ways. To make this more acceptable, applicable, culturally appropriate, and evidence-based, there is a strong need to conduct targeted research efforts such as systematic identification of multi-level factors associated with CVH and delivery of VHI in different settings (e.g., hospital, rural). Lessons learned from our model and intervention can assist in implementation, adoption of intervention, allowing policymakers, researchers, and stakeholders to consider the strengths and challenges of our model to implement a CVH intervention in their countries and other parts of India.

### 4.6. Strengths and limitations

To the best of our knowledge, our model is the first of its kind to address CVH among perinatal women in LMICs. The model provides a better perspective in terms of medical ethics, informed decision, appropriateness, and acceptability. The model has been developed considering the stakeholders' perspectives and using the WHO implementation toolkit (11). This model helps to understand the individual level determinants of CVH and can be instrumental in bringing positive changes in vaccination uptake within the different framework of the Indian Health care systems (private and government, urban and rural, primary, secondary, and tertiary). The content of CVH intervention was developed with the goal of compatibility across implementation and delivery systems, which helped to improve their scalability potential. The CVH Intervention and telephonic reminders will improve adherence and outcomes. In terms of limitations, it is worth noting that the consensus method can be prone to subjectivity and opinion bias.

## 5. Conclusion

The CCPP model is a suggested new stepped-care model centered on primary HCWs that can be useful in addressing CVH among perinatal women comprehensively and effectively in LMICs. Implementing this model in the existing COVID-19 vaccination centers, antenatal clinics, postnatal clinics, and immunization clinics may be a more feasible, sustainable, and acceptable approach to increasing capacity for CVH screening and intervention. Future research should look into the barriers and enablers affecting the implementation of the CCPP model in real-world settings.

## Data availability statement

The raw data supporting the conclusions of this article will be made available by the authors, without undue reservation.

## Ethics statement

The studies involving human participants were reviewed and approved by Institutional Ethics Committee, BKL Walawalkar Rural Medical College, Kasturba Medical College, Manglore, Lady Hardinge Medical College, New Delhi. The patients/participants provided their written informed consent to participate in this study.

## Author contributions

RR, PR, and PKuk conceived the project and collected the data. RR wrote the first draft of the manuscript. RR, PR, MP, KP, SP, and PKuk wrote sections of the manuscript. All authors contributed to manuscript revision, read, and approved the submitted version.
